# Role of Thr399Ile and Asp299Gly polymorphisms of toll-like receptor-4 gene in acute dental abscess

**DOI:** 10.4317/jced.53190

**Published:** 2017-02-01

**Authors:** Ebrahim Miri-Moghaddam, Narges Farhad-Mollashahi, Elnaz Baghaee, Ali Bazi, Yasaman Garme

**Affiliations:** 1 Genetics of Non-Communicable Disease Research Center & Department of Genetics, Faculty of Medicine, Zahedan University of Medical Sciences, Zahedan, Iran; 2 Oral and Dental Diseases Research Center, Department of Endodontics, Faculty of Dentistry, Zahedan University of Medical Sciences, Zahedan, Iran; 3 Faculty of Dentistry, Zahedan University of Medical Sciences, Zahedan, Iran; 4 Faculty of Allied Medical Sciences, Zabol University of Medical Sciences, Zabol, Iran; 5 Cellular and Molecular Research Center, Zahedan University of Medical Sciences, Zahedan, Iran

## Abstract

**Background:**

Apical Periodontitis (AP) is an inflammatory disease that affects the tissues surrounding the root end of a tooth. The disease which is caused by endodontic infections presents in different clinical ways including development of an acute abscess. Recent studies have provided information suggesting role of a multitude of factors in pathogenesis of acute apical abscess (AAA). In this case-control study, our goal was to evaluate the frequency and potential role of two common polymorphisms of toll like receptor-4 (TLR-4) gene; Thr399Ile (1196 C>T) and Asp299Gly (+896 A>G), in 50 patients with AAA as cases and 50 patients with asymptomatic apical periodontitis (AAP) as controls.

**Material and Methods:**

Saliva sample containing mucosal epithelial cells was used for DNA extraction. Polymorphisms were detected by Tetra-ARMS (Amplification Refractory Mutation System) PCR method. Statistical analyses were carried out in SPSS 21 software.

**Results:**

Homozygous wild type (CC) and heterozygous (CT) genotypes of Thr399Ile polymorphism were detected in 84% and 16% of AAA patients respectively. In controls, respective ratios were 94% (CC) and 6% (CT). Observed difference was not statistically significant (*P*>0.05) for distribution of these genotypes. The mutant homozygous (TT) genotype of this polymorphism was identified in neither of the participants. Overall, T allele frequency was obtained 8% in AAA and 3% in AAP (OR=2.6, 95% CI; 0. 6-10.6, *p*>0.05). For Asp299Gly polymorphism, no individual was detected with the mutant allele in case or control groups.

**Conclusions:**

Our results indicated a possible role for Thr399Ile polymorphism in acute presentations of abscess in AAA. However, the impact of this polymorphism needs to be more assessed in future studies.

** Key words:**Genetic polymorphism, periapical abscess, periapical periodontitis, toll-like receptor 4.

## Introduction

Apical abscess is an inflammatory process in the peri-radicular tissues caused by biofilms in the necrotic root canal systems (1). It is usually localized intraorally, but in some cases the apical abscess may spread and result in severe complications or even mortality. The reasons why dental root canal infections can become symptomatic and evolve to severe spreading and sometimes life-threatening abscesses remains elusive. Recent studies in the fields of microbiology and immunology have provided information regarding pathogenesis of symptomatic apical periodontitis, including its most severe form, the acute apical abscess (AAA) (1,2). Although mediators involved in clinical picture of apical periodontitis (AP) are less certain, role of immune system components has been suggested (3).

Innate immunity system, as the main mediator of inflammation, recruits specific pattern recognition receptors (PRR) capable of identifying conserved pathogen-associated molecular patterns within microorganisms. Toll like receptors (TLRs) are the most studied subtypes of PRRs. Ten TLRs covering a wide range of antigenic determinants have been recognized in humans (4). Among these, TLR-4 is a well-known member of TLR family residing in plasma membrane of multiple types of immune cells. TLR-4 is the main receptor responsible for recognition of lipopolysacharid (LPS), the dominant component of gram-negative bacteria cell wall (4,5). TLR-4 activation leads to production of inflammatory cytokines such as tumor necrosis factor- α (TNF-α), interleukin-6 (IL-6), IL-8, and also to induction of Nuclear factor κB(NF- κB ), an inflammatory transcription factor (6). Interestingly, altered expression of TLR-4 has been described in periodontitis which suggests a role for this receptor in pathogenesis of AP (7).

Single nucleotide polymorphisms (SNPs) in the genes of TLRs or cytokines have been known as important determinants modulating the inflammatory reactions and the balance of pro/anti-inflammatory state in AP (8). Susceptibility to periodontitis has been associated with polymorphisms in the genes of pro-inflammatory cytokines; IL-4 and IL-10 (9), IL-6 (10) and IL-8 (11). As well, considering that LPS is the main ligand of TLR-4, SNP within TLR-4 gene has been reported to increase the susceptibility to bacterial infections (4). In parallel, polymorphisms of TLR-4 and its co-receptor (CD 14) have been found to be related to periodontitis (12). The two most studied TLR-4 polymorphisms are Thr399Ile (rs4986791) and Asp299Gly (rs4986790). These two polymorphisms interfere with TLR-4 signal transduction, and therefore potentially affect the immune response to microbial invasions and susceptibility to infections (13,14). In some studies, frequency of Asp299Gly and Thr399Ile polymorphisms has been reported to be significantly higher in patients with periodontitis (15,16).

Studies of genetic polymorphisms in AP are very limited, and only eight eligible studies have been compiled in a recent comprehensive review (17). Besides, there was no study on the role of Thr399Ile and Asp299Gly polymorphisms of TLR-4 in AP patients. In present study, we aimed to assess the significance of the Asp299Gly and Thr399Ile polymorphisms in patients with AAA and asymptomatic apical periodontitis (AAP) in South-East of Iran.

## Material and Methods

-Population

The subjects were selected from the city of Zahedan in South-East of Iran. Patients and controls were chosen from individuals visiting therapeutic center, Faculty of Dental medicine, Zahedan University of Medical Sciences within 2014. The study was approved by Ethical committee of Research of the University, and the participants were also asked to sign an informed consent. Demographical data such as age, sex, vacancy, education, as well as teeth type and jaw position were obtained by interviews and oral examinations. The participants were sub classified according to radiographic findings and clinical manifestations into two groups: 50 patients with AAA (as case group) and 50 ethnic, age and sex matched patients with AAP (as control group). Inclusion criteria for AAA cases consisted of moderate to severe signs and symptoms along with soft tissue swelling with or without systemic manifestations such as fever, malaise, headache and lymphadenopathy. This condition is characterized by pulp necrosis and slightly thickened periodontal ligament space to a radiolucent lesion in radiographs. Patients in the control group exhibited pulp necrosis with no or only mild signs and symptoms and without previous exacerbation, which included radiographically thickening of the periodontal ligament space compatible with periapical lesion. The patients of both groups were from the same geographic area (8,18).

-Exclusion criteria

Followings were considered as exclusion criteria in both AAA and AAP groups: prior endodontic treatment, teeth with an intra or extra oral sinus tract and subjects with systemic diseases, severe bleeding disorders, history of corticosteroids or NSAIDs consumption, HIV or chemo or radiotherapy.

-Sample collection

Saliva sample containing oral epithelial cells were exploited for DNA extraction. Exfoliated mucosal epithelial cells were expectorated into sterile micro tubes, and then the samples were taken to the laboratory for DNA extraction.

-DNA extraction and polymorphisms genotyping

DNA was obtained as previously described (19).We applied Tetra-ARMS (Amplification Refractory Mutation System) PCR method for identification of the Thr399Ile and Asp299Gly polymorphisms. In this method, four primers (two outers and two inners) are simultaneously introduced into one PCR reaction. This method provides highly specific and cost-effective allele-specific amplifications. Sequences of primers and length of PCR products have been presented in  Table 1.

**Table 1:**
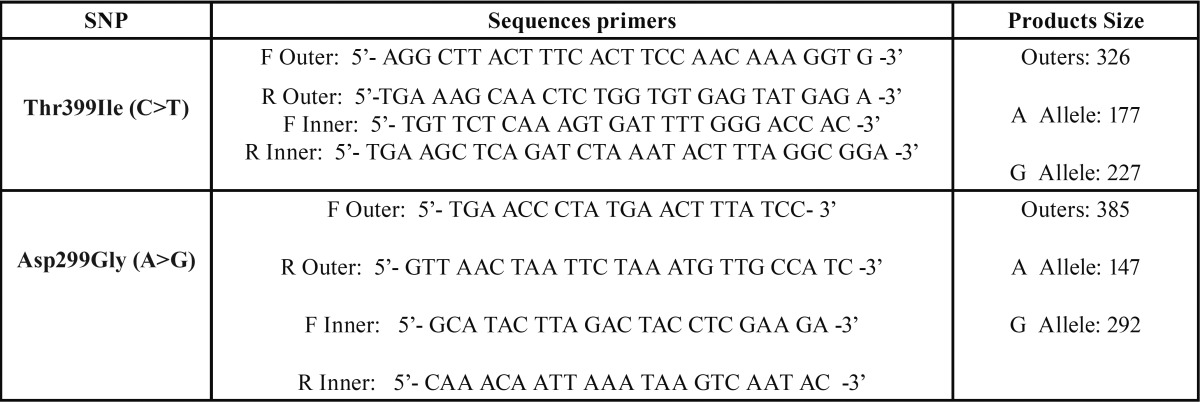
Primer sequences for detection of Thr399Ile and Asp299Gly polymorphisms by tetra-ARMS method.

-PCR reaction

PCR reaction was run at net reaction volume of 25 μl. Genomic DNA (50 ng), 1 μl of each primer (0.1 pMol) and 12.5 μl Taq DNA polymerase master mix (Pishgam, Iran) were admixed in a single reaction tube, and the net volume was reached by adding distilled water. PCR was initiated with 5 min of denaturation phase at 94. The reaction was continued with 30 (for Thr399Ile) and 31 (for Asp299Gly) thermal cycles of denaturation (5 min at 94), annealing (30 seconds at 60 for Thr399Ile and 64 for Asp299Gly) and extension (30 seconds at 72). At last, a final 5 minutes of extension phase at 72 was allowed. The PCR products were electrophoresed on 2% Agarose gel, and then photographed using green viewer dye and UV light.

-Statistical analyses

All analyses were performed by SPSS 21 software. Chi-square test was exploited for seeking a possible association between AAA with categorical variables such as sex, position of jaw or type of the teeth involved. When appropriate, fisher exact test was considered for reporting p values of associations between these categorical variables and the clinical outcome. Independent samples t-student test was employed to evaluate if there was a significant difference between mean age of AAA and AAP groups. For conduction a risk association analysis, logistic regression was the statistical procedure of choice. A dominant model of logistic regression was conducted for assessing risk of genotypes. Statistical significance threshold (*P* value) was considered as 0.05.

## Results

There were no significant differences between case and control groups considering demographical features ( Table 2).

**Table 2:**
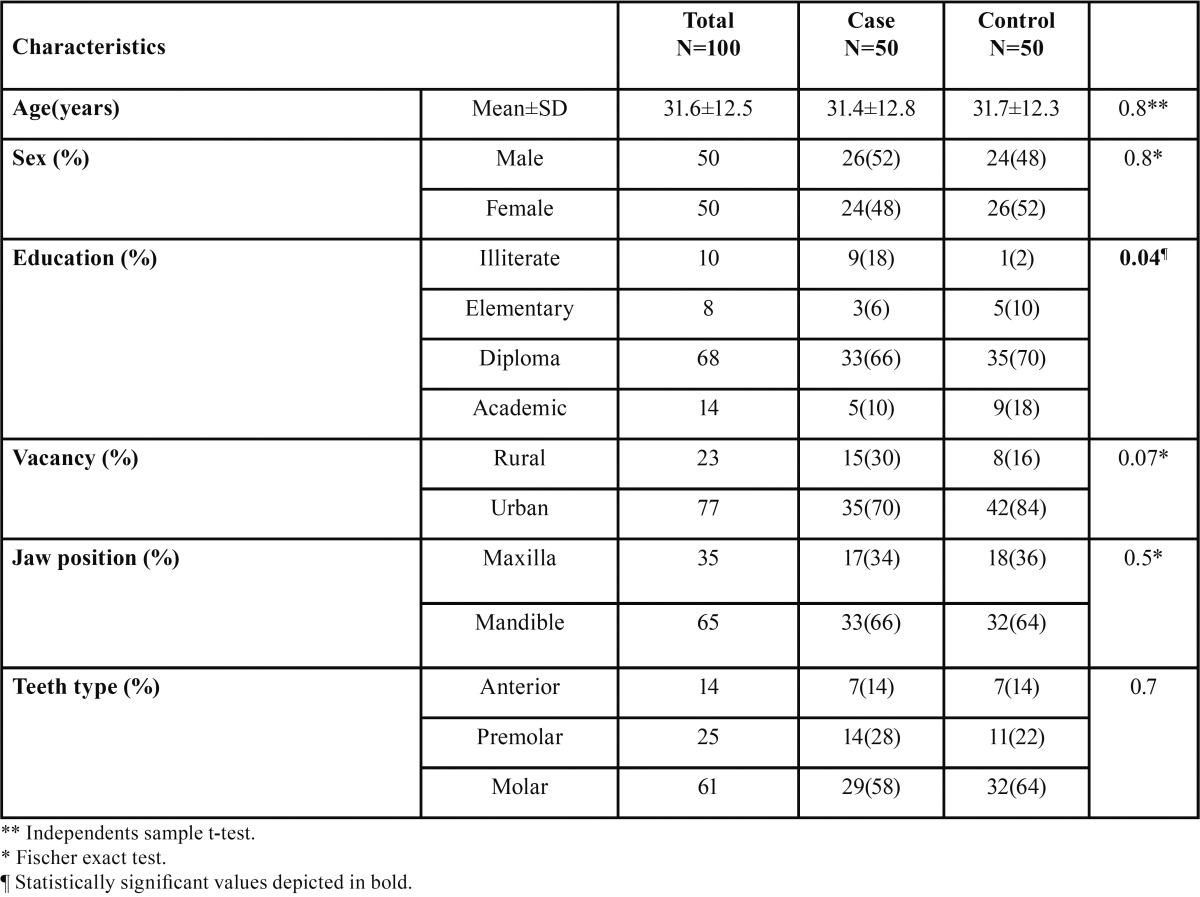
Demographical and clinical features of AAA (case) and AAP (control) patients.

Homozygous wild type (CC) of Thr399Ile (1196 C>T) polymorphism was the dominant genotype in both AAA and AAP patients (respective percentages of 84% and 94%). Heterozygous (CT) genotype was also identified in 16% and 6% of symptomatic and non-symptomatic patients respectively ( Table 3). Frequencies of mutant (T) allele of Thr399Ile were 8% and 3% in cases and controls respectively; however, the difference was not statistically significant. Homozygous wild type (AA) genotype of Asp299Gly (+896 A>G) polymorphism was the sole combination discovered in the all participants. Furthermore homozygous genotype of mutant allele was not found in neither of the polymorphisms nor for AAA or AAP patients.

**Table 3:**
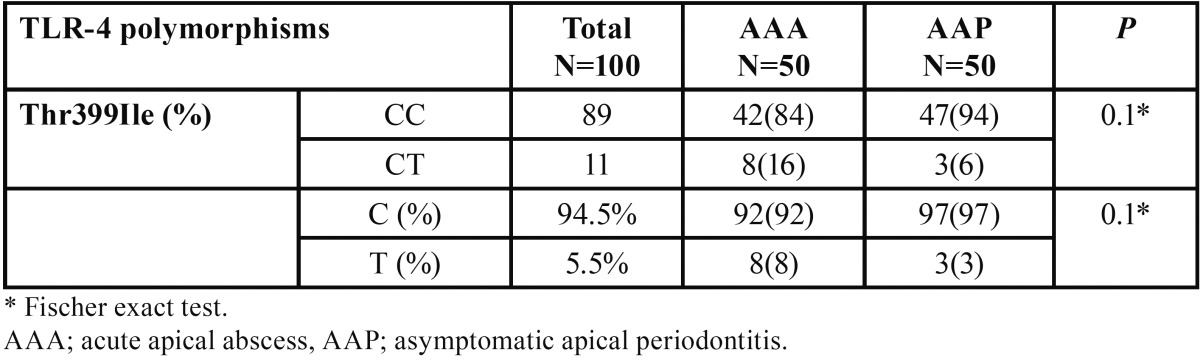
Genotype and allele frequency of Thr399Ile polymorphism of TLR-4 in AAA and AAP patients.

Logistic regression analysis revealed that mutant allele (T) of Thr399Ile polymorphism may be a risk factor for acute abscess formation (OR= 2.6, 95 CI: 0.6-10.6). Likewise, heterozygous genotype (CT) of this polymorphism was associated with higher rate of AAA occurrence (OR= 2.9, *P*>0.05).  Table 4 shows respective ORs for evaluated variables.

**Table 4:**
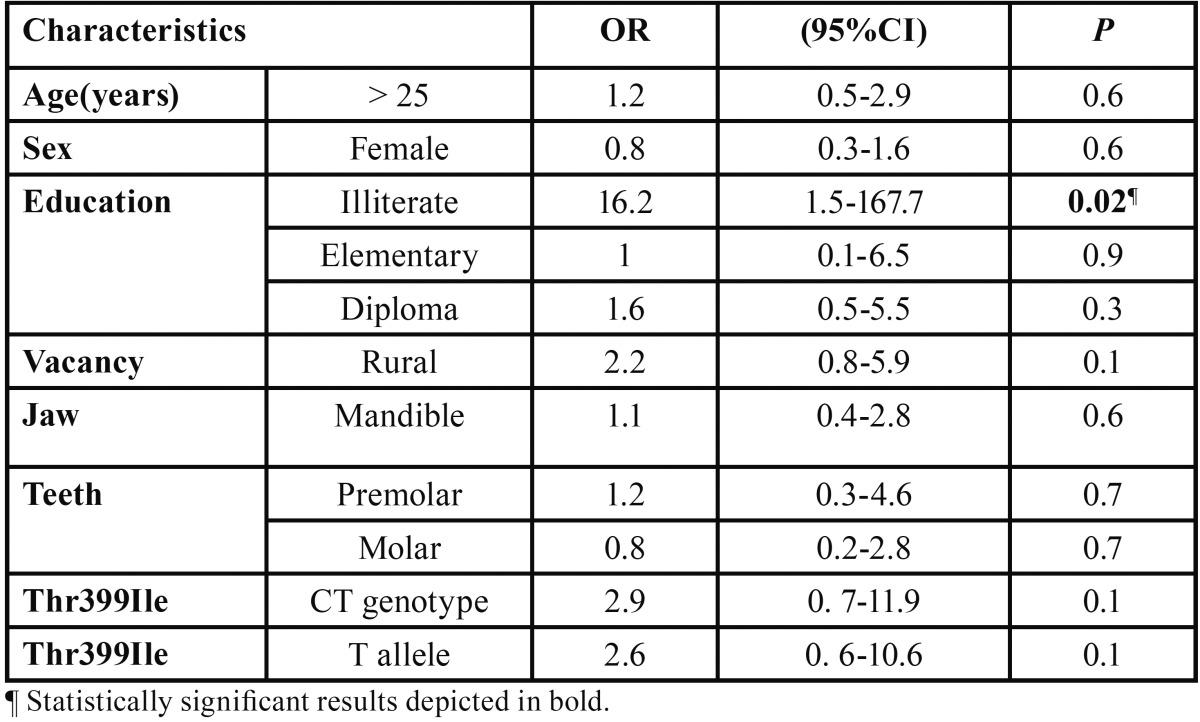
Logistic regression analysis performed for demographic and genetic characteristics for risk of AAA development.

## Discussion

In present study, we assessed a potential relationship between two well-known TLR-4 polymorphisms; Thr399Ile and Asp299Gly with two clinical forms of periapical disease; AAA and AAP. TLR-4 is an important member of antigen recognition compartment of innate immunity system. As well, this receptor participates in adhesion reactions involved in transmigration of leukocyte to infected apical tissues (20). In current work, we found that distribution of Thr399Ile and Asp299Gly polymorphisms was not significantly different between AAA and AAP individuals. However, minor allele (T) of Thr399Ile polymorphism demonstrated a higher frequency in AAA than AAP patients (OR=2.9,  Table 4).

TLR-4 is the main receptor for LPS of gram negative bacteria. Both Asp299Gly and Thr399Ile polymorphisms have been described to reduce binding and signaling capacity of TLR-4 after exposure to LPS antigenic determinants (21). Also, presence of these polymorphisms elevated the risk of vigorous tissue infections by gram negative bacteria (22). In addition, it’s been reported that gram negative bacteria are among the main identified microorganisms in AP, and specially higher load of these bacteria are present in AAA patients than AAP (23). Considering this in mind, higher risk of AAA in presence of Thr399Ile polymorphism may be explainable by lower signaling activity of TLR-4 following of bacterial invasion. Nevertheless, mutant allele of Asp299Gly polymorphism was not found in our studied population; and therefore we were unable to carry out a risk stratification analysis for this polymorphism.

Few studies are available evaluating the role of TLR-4 polymorphisms in AP, and especially we found no study on Thr399Ile and Asp299Gly polymorphisms. In study of Rôças *et al.* (24), +896A>G polymorphism (known to cause less ligand binding efficacy) of TLR-4 showed no impact on persistent AP in Brazilians. Polymorphisms of pro inflammatory mediators and Fcү receptor have been described to affect clinical course of AP (8,25). Also, polymorphisms of tissue remodeling enzymes have been reported as potential effectors of clinical features in AP (26). High producer alleles of IL-1β, IL-6, and IL-8 pro inflammatory cytokines have also been related to elevated risk of acute dental abscess (8,18). On the other hand, a relationship has been identified between low producer allele of TNF-α and symptomatic abscess (18). In oppose to this, there are also studies which have found no link between clinical forms of AP and polymorphisms of IL-1β and TNF-α (8).

Previous reports have yielded somehow inconsistent conclusions regarding association of TLR-4 polymorphisms and risk of marginal periodontitis. Marginal periodontitis are similar to AP in many aspects including bacterial profiles and involved immune responses, and only differ in few features including patterns of bacterial colony formation (24). TLR-4 polymorphisms of Asp299Gly and Thr399Ile have not been associated with aggressive periodontitis (AgP) or chronic periodontitis in previous reports (27-29). In study of Schröder *et al.* (15), TLR-4 Thr399Ile polymorphism has been related to a significantly higher risk of CP but not AgP. Considering biologic similarities of marginal periodontitis and AP, higher OR of Thr399Ile polymorphism for AP observed here is in line with prior observations in marginal periodontitis; however, this needs to be clarified in larger population-based studies.

Failure of our results reaching a statistical significant threshold may be in part related to relatively small number of our participants; nevertheless, our sample was greater than multiple genuine studies in AP (24,30). Also, taking into consideration of environmental factors is of critical importance. Furthermore, AP clinical picture can potentially be modulated by the function of other immune components participating in inflammatory response. In addition to the genetic contributors, considering acquired factors in risk association studies on AP is recommended. Conclusively, taking in mind of an interactive approach between genetic and acquired determinants may provide a more comprehensive knowledge of AP pathogenesis.

Our result demonstrated higher occurrence of TLR-4 Thr399Ile polymorphism in AAA than AAP patients suggesting a triggering role for variant allele of this polymorphism for symptomatic form of AP. Nevertheless studies on larger sample populations are encouraged in order to emerge any significant results.
